# Dichlorido[*N*,*N*-diethyl-*N*′-(2-pyridyl­methyl­ene)ethane-1,2-diamine]mercury(II)

**DOI:** 10.1107/S1600536810000103

**Published:** 2010-01-09

**Authors:** Young-Inn Kim, Hoe-Joo Seo, Ji-Hoon Kim, You-Soon Lee, Sung Kwon Kang

**Affiliations:** a Department of Chemistry Education, Interdisciplinary Program of Advanced Information and Display Materials, and Center for Plastic Information Systems, Pusan National University, Busan 609-735, Republic of Korea; b Department of Chemistry, Chungnam National University, Daejeon 305-764, Republic of Korea

## Abstract

The Hg atom in the title compound, [HgCl_2_(C_12_H_19_N_3_)], adopts a distorted trigonal-bipyramidal geometry, being ligated by two Cl atoms and three N atoms of the *N*,*N*-diethyl-*N*′-(2-pyridylmethyl­ene)ethane-1,2-diamine ligand. The dihedral angle between the HgN_3_ and HgCl_2 _least-squares planes is 88.6 (1)°. The Hg—N distances  including the pyridine N and the ammonium N atom are about 0.20 Å longer than the Hg—N distance including the imino N atom.

## Related literature

For general background to luminescent mercury compounds, see: Elena *et al.* (2006 ▶); Durantaye *et al.* (2006 ▶); Fan *et al.* (2009 ▶). For the syntheses and structures of these compounds, see: Kim *et al.* (2008 ▶); Seo *et al.* (2009 ▶).
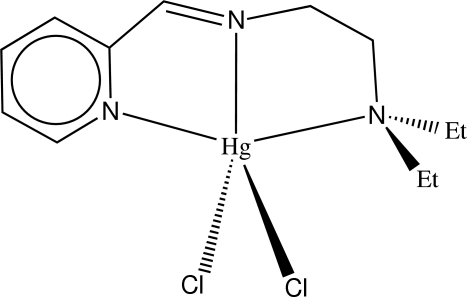


## Experimental

### 

#### Crystal data

[HgCl_2_(C_12_H_19_N_3_)]
*M* *_r_* = 476.79
Monoclinic, 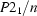

*a* = 8.0028 (5) Å
*b* = 16.6507 (9) Å
*c* = 12.4541 (8) Å
β = 101.630 (5)°
*V* = 1625.47 (17) Å^3^
*Z* = 4
Mo *K*α radiation
μ = 9.79 mm^−1^
*T* = 295 K
0.27 × 0.24 × 0.23 mm

#### Data collection

Bruker SMART CCD area-detector diffractometer
Absorption correction: multi-scan (*SADABS*; Bruker, 2002 ▶) *T*_min_ = 0.085, *T*_max_ = 0.102
17026 measured reflections
4039 independent reflections
3124 reflections with *I* > 2σ(*I*)
*R*_int_ = 0.029

#### Refinement

*R*[*F* ^2^ > 2σ(*F* ^2^)] = 0.026
*wR*(*F* ^2^) = 0.059
*S* = 1.04
4039 reflections
163 parameters
H-atom parameters constrained
Δρ_max_ = 0.71 e Å^−3^
Δρ_min_ = −0.92 e Å^−3^

### 

Data collection: *SMART* (Bruker, 2002 ▶); cell refinement: *SAINT* (Bruker, 2002 ▶); data reduction: *SAINT*; program(s) used to solve structure: *SHELXS97* (Sheldrick, 2008 ▶); program(s) used to refine structure: *SHELXL97* (Sheldrick, 2008 ▶); molecular graphics: *ORTEP-3 for Windows* (Farrugia, 1997 ▶); software used to prepare material for publication: *WinGX* (Farrugia, 1999 ▶).

## Supplementary Material

Crystal structure: contains datablocks global, I. DOI: 10.1107/S1600536810000103/tk2606sup1.cif

Structure factors: contains datablocks I. DOI: 10.1107/S1600536810000103/tk2606Isup2.hkl

Additional supplementary materials:  crystallographic information; 3D view; checkCIF report

## Figures and Tables

**Table 1:** Selected bond lengths (Å)

Hg1—Cl1	2.4088 (11)
Hg1—Cl2	2.4431 (11)
Hg1—N1	2.540 (3)
Hg1—N8	2.336 (3)
Hg1—N11	2.544 (3)

**Fig. 1.**
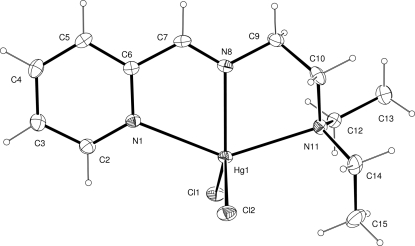
Molecular structure of (I), showing the atom-numbering scheme and 30% probability ellipsoids.

## supplementary crystallographic information

### Comment

Much attention has been paid to the design and synthesis of luminescent mercury compounds for the detection and extraction of the mercury (Elena *et al.*, 2006; Durantaye *et al.*, 2006), among which, Hg(II) complexes with pyridine-containing ligands are of importance for their high luminescent efficiency (Fan *et al.*, 2009). Recently, we reported Hg(II) compounds with bis(2-pyridylmethyl)amine (Kim *et al.*, 2008) and with benzyl(2-pyridylmethyl)amine (Seo *et al.*, 2009) as a development of blue fluorescent materials. In this work, we prepared a Hg(II) complex with *N*,*N*-diethyl-*N*'-pyridine-2-ylmethylene-ethene-1,2-diamine (depmed), and its structure and luminescent properties were investigated.

In the title compound, (I), the Hg atom is 5-coordinated by two Cl atoms and three N atoms of the tridentate depmed ligand. The coordination geometry around Hg atom is based on a distorted trigonal bipyramid with the equatorial plane defined by N8, Cl1, and Cl2 atoms, with the other N atoms occupying axial positions. The dihedral angle between the least-squares planes through the N1/N8/N11/Hg atoms and that through the HgCl_2_ atoms is 88.6 (1)°; the bond angle of N1—Hg—N11 is 139.2 (1)°. The Hg–N1 and Hg–N11 bond distances are each about 0.20Å longer than the Hg–N8 bond distance, Table 1.

The free ligand (depmed) showed strong blue (λ_max,PL_ = 491 nm in DMF) fluorescent emissions upon 280 nm excitation, while Hg(depmed)Cl_2_ displayed two blue emission (λ_max,PL_ = 309 and 389 nm in DMF) which was tentatively assigned to be an intraligand (IL) ^1^π-π^*^ transition. The PL quantum yield (*f*) *versus* 9,10-diphenylanthracene was measured to be 0.29% and 0.04% for free ligand (depmed) and Hg(depmed)Cl_2_, respectively.

### Experimental

All of the reagents and solvents were purchased from Aldrich and used without further purification. The *N*,*N*-diethyl-*N*'-pyridine-2-ylmethylene-ethene-1,2-diamine (*L*) was synthesized by reacting *N*,*N*-diethyl-ethylenediamine (15 mmol) and 2-pyridinecarboxaldehyde (15 mmol) in methanol (50 ml). The mixture was stirred for 3 h at room temperature and the solution was evaporated to dryness. The residue was extracted with dichloromethane to give depmed as yellow oil. A solution depmed (5 mmol) in methanol (15 ml) was added slowly to a solution of mercuric chloride (5 mmol) in methanol (15 ml). The mixture was stirred for 12 h at room temperature. The resultant precipitate was collected by filtration and washed several times with cool methanol. The precipitate was dried over vacuum in an oven at room temperature. The crystals were obtained by slow evaporation in a methanol solution. Yield: 53%. Anal. Calcd. for C_12_H_19_N_3_Cl_2_Hg: C, 30.23; H, 4.02; N, 8.81. Found: C, 29.97; H, 4.21; N, 8.76. ^1^H-NMR (300 MHz, d_6_-DMSO) δ; 8.95 (1*H*, d, J=4.2 Hz), 8.57 (1*H*, s), 7.97 (1*H*, t, J=7.8 Hz), 7.62–7.68 (2*H*, m), 3.82 (2*H*, t, J=6.5 Hz), 2.94–3.08 (6*H*, m), 1.19 (6*H*, s).

### Refinement

All H atoms were positioned geometrically and refined using a riding model, with C—H = 0.93 - 0.97 Å, and with *U*_iso_(H) = 1.2*U*_eq_(C) for aromatic- and methylene-H, and 1.5*U*_eq_(C) for methyl-H atoms.

### Crystal data

**Table:**

[HgCl_2_(C_12_H_19_N_3_)]	*F*(000) = 904
*M**_r_* = 476.79	*D*_x_ = 1.948 Mg m^−^^3^
Monoclinic, *P*2_1_/*n*	Mo *K*α radiation, λ = 0.71073 Å
Hall symbol: -P 2yn	Cell parameters from 5949 reflections
*a* = 8.0028 (5) Å	θ = 2.5–26.4°
*b* = 16.6507 (9) Å	µ = 9.79 mm^−^^1^
*c* = 12.4541 (8) Å	*T* = 295 K
β = 101.630 (5)°	Block, colourless
*V* = 1625.47 (17) Å^3^	0.27 × 0.24 × 0.23 mm
*Z* = 4	

### Data collection

**Table:**

Bruker SMART CCD area-detector diffractometer	3124 reflections with *I* > 2σ(*I*)
φ and ω scans	*R*_int_ = 0.029
Absorption correction: multi-scan (*SADABS*; Bruker, 2002)	θ_max_ = 28.3°, θ_min_ = 2.1°
*T*_min_ = 0.085, *T*_max_ = 0.102	*h* = −10→10
17026 measured reflections	*k* = −22→22
4039 independent reflections	*l* = −14→16

### Refinement

**Table:**

Refinement on *F*^2^	0 restraints
Least-squares matrix: full	H-atom parameters constrained
*R*[*F*^2^ > 2σ(*F*^2^)] = 0.026	*w* = 1/[σ^2^(*F*_o_^2^) + (0.026*P*)^2^ + 0.5659*P*] where *P* = (*F*_o_^2^ + 2*F*_c_^2^)/3
*wR*(*F*^2^) = 0.059	(Δ/σ)_max_ = 0.001
*S* = 1.04	Δρ_max_ = 0.71 e Å^−^^3^
4039 reflections	Δρ_min_ = −0.92 e Å^−^^3^
163 parameters	

### Special details

**Table:**

Geometry. All e.s.d.'s (except the e.s.d. in the dihedral angle between two l.s. planes) are estimated using the full covariance matrix. The cell e.s.d.'s are taken into account individually in the estimation of e.s.d.'s in distances, angles and torsion angles; correlations between e.s.d.'s in cell parameters are only used when they are defined by crystal symmetry. An approximate (isotropic) treatment of cell e.s.d.'s is used for estimating e.s.d.'s involving l.s. planes.

### Fractional atomic coordinates and isotropic or equivalent isotropic displacement parameters (Å^2^)

**Table:**

	*x*	*y*	*z*	*U*_iso_*/*U*_eq_	
Hg1	0.208069 (19)	0.190628 (9)	0.561325 (12)	0.05266 (7)	
Cl1	0.04834 (16)	0.20511 (8)	0.70474 (10)	0.0766 (3)	
Cl2	0.06896 (15)	0.15227 (8)	0.37542 (9)	0.0756 (3)	
N1	0.3474 (4)	0.0549 (2)	0.6108 (3)	0.0533 (8)	
C2	0.2740 (6)	−0.0156 (3)	0.6201 (4)	0.0654 (11)	
H2	0.1555	−0.0177	0.6057	0.078*	
C3	0.3629 (7)	−0.0862 (3)	0.6499 (4)	0.0697 (12)	
H3	0.3058	−0.1342	0.6553	0.084*	
C4	0.5359 (7)	−0.0830 (3)	0.6708 (4)	0.0731 (13)	
H4	0.5996	−0.1292	0.6915	0.088*	
C5	0.6169 (6)	−0.0108 (3)	0.6612 (3)	0.0659 (11)	
H5	0.7353	−0.0078	0.6745	0.079*	
C6	0.5184 (5)	0.0570 (2)	0.6313 (3)	0.0527 (9)	
C7	0.5949 (5)	0.1352 (3)	0.6185 (3)	0.0584 (10)	
H7	0.713	0.1391	0.6283	0.07*	
N8	0.5056 (4)	0.19763 (19)	0.5946 (3)	0.0554 (8)	
C9	0.5810 (6)	0.2749 (3)	0.5783 (4)	0.0706 (12)	
H9A	0.6906	0.2671	0.5578	0.085*	
H9B	0.599	0.3057	0.6458	0.085*	
C10	0.4618 (7)	0.3196 (2)	0.4886 (4)	0.0721 (14)	
H10A	0.5111	0.3715	0.4782	0.087*	
H10B	0.451	0.2899	0.4206	0.087*	
N11	0.2900 (5)	0.33175 (19)	0.5129 (3)	0.0545 (8)	
C12	0.2909 (6)	0.3827 (2)	0.6098 (3)	0.0597 (10)	
H12A	0.3544	0.3549	0.6736	0.072*	
H12B	0.1743	0.388	0.6197	0.072*	
C13	0.3658 (7)	0.4666 (3)	0.6073 (4)	0.0860 (15)	
H13A	0.3604	0.4942	0.6742	0.129*	
H13B	0.3016	0.496	0.5463	0.129*	
H13C	0.4825	0.4627	0.5996	0.129*	
C14	0.1738 (7)	0.3608 (3)	0.4138 (4)	0.0836 (15)	
H14A	0.1783	0.3241	0.354	0.1*	
H14B	0.2134	0.4128	0.3942	0.1*	
C15	−0.0096 (8)	0.3686 (4)	0.4267 (5)	0.112 (2)	
H15A	−0.0782	0.3881	0.3595	0.168*	
H15B	−0.0157	0.4057	0.4849	0.168*	
H15C	−0.051	0.3171	0.444	0.168*	

### Atomic displacement parameters (Å^2^)

**Table:**

	*U*^11^	*U*^22^	*U*^33^	*U*^12^	*U*^13^	*U*^23^
Hg1	0.04281 (9)	0.06545 (11)	0.05013 (10)	−0.00279 (7)	0.01033 (7)	0.00321 (7)
Cl1	0.0616 (7)	0.1078 (9)	0.0680 (7)	−0.0054 (6)	0.0312 (6)	−0.0045 (6)
Cl2	0.0637 (7)	0.0923 (8)	0.0615 (7)	−0.0041 (6)	−0.0097 (6)	−0.0122 (6)
N1	0.0474 (18)	0.065 (2)	0.0478 (18)	0.0010 (15)	0.0114 (15)	0.0030 (15)
C2	0.060 (3)	0.076 (3)	0.066 (3)	−0.002 (2)	0.025 (2)	0.011 (2)
C3	0.082 (3)	0.070 (3)	0.065 (3)	−0.001 (2)	0.033 (3)	0.011 (2)
C4	0.092 (4)	0.068 (3)	0.062 (3)	0.023 (3)	0.024 (3)	0.014 (2)
C5	0.057 (3)	0.080 (3)	0.059 (3)	0.020 (2)	0.009 (2)	0.000 (2)
C6	0.051 (2)	0.067 (2)	0.040 (2)	0.0014 (19)	0.0081 (17)	−0.0043 (17)
C7	0.037 (2)	0.080 (3)	0.058 (2)	0.003 (2)	0.0084 (18)	−0.014 (2)
N8	0.0451 (18)	0.066 (2)	0.057 (2)	−0.0078 (15)	0.0133 (16)	−0.0080 (16)
C9	0.052 (3)	0.069 (3)	0.095 (4)	−0.008 (2)	0.025 (3)	−0.004 (3)
C10	0.089 (4)	0.063 (3)	0.078 (3)	−0.019 (2)	0.050 (3)	−0.005 (2)
N11	0.065 (2)	0.0620 (19)	0.0372 (17)	−0.0039 (16)	0.0116 (16)	0.0066 (14)
C12	0.068 (3)	0.066 (3)	0.047 (2)	0.003 (2)	0.016 (2)	0.0030 (19)
C13	0.115 (4)	0.070 (3)	0.078 (3)	−0.006 (3)	0.031 (3)	−0.012 (3)
C14	0.115 (5)	0.080 (3)	0.048 (3)	−0.004 (3)	−0.003 (3)	0.019 (2)
C15	0.099 (5)	0.117 (5)	0.102 (4)	0.024 (4)	−0.024 (4)	0.018 (4)

### Geometric parameters (Å, °)

**Table:**

Hg1—Cl1	2.4088 (11)	C9—H9A	0.97
Hg1—Cl2	2.4431 (11)	C9—H9B	0.97
Hg1—N1	2.540 (3)	C10—N11	1.480 (6)
Hg1—N8	2.336 (3)	C10—H10A	0.97
Hg1—N11	2.544 (3)	C10—H10B	0.97
N1—C2	1.328 (5)	N11—C14	1.470 (5)
N1—C6	1.341 (5)	N11—C12	1.473 (5)
C2—C3	1.385 (6)	C12—C13	1.524 (6)
C2—H2	0.93	C12—H12A	0.97
C3—C4	1.357 (6)	C12—H12B	0.97
C3—H3	0.93	C13—H13A	0.96
C4—C5	1.384 (6)	C13—H13B	0.96
C4—H4	0.93	C13—H13C	0.96
C5—C6	1.383 (6)	C14—C15	1.514 (8)
C5—H5	0.93	C14—H14A	0.97
C6—C7	1.462 (6)	C14—H14B	0.97
C7—N8	1.262 (5)	C15—H15A	0.96
C7—H7	0.93	C15—H15B	0.96
N8—C9	1.452 (5)	C15—H15C	0.96
C9—C10	1.511 (7)		
			
N8—Hg1—Cl1	122.54 (9)	C10—C9—H9B	109.9
N8—Hg1—Cl2	115.75 (9)	H9A—C9—H9B	108.3
Cl1—Hg1—Cl2	121.35 (4)	N11—C10—C9	113.0 (3)
N8—Hg1—N1	67.63 (11)	N11—C10—H10A	109
Cl1—Hg1—N1	100.47 (8)	C9—C10—H10A	109
Cl2—Hg1—N1	95.24 (8)	N11—C10—H10B	109
N8—Hg1—N11	72.16 (12)	C9—C10—H10B	109
Cl1—Hg1—N11	106.49 (8)	H10A—C10—H10B	107.8
Cl2—Hg1—N11	96.14 (8)	C14—N11—C12	113.3 (4)
N1—Hg1—N11	139.24 (11)	C14—N11—C10	109.3 (4)
C2—N1—C6	117.3 (4)	C12—N11—C10	113.2 (4)
C2—N1—Hg1	128.9 (3)	C14—N11—Hg1	110.7 (3)
C6—N1—Hg1	113.9 (3)	C12—N11—Hg1	107.2 (2)
N1—C2—C3	124.1 (4)	C10—N11—Hg1	102.5 (2)
N1—C2—H2	117.9	N11—C12—C13	116.6 (3)
C3—C2—H2	117.9	N11—C12—H12A	108.1
C4—C3—C2	117.9 (4)	C13—C12—H12A	108.1
C4—C3—H3	121.1	N11—C12—H12B	108.1
C2—C3—H3	121.1	C13—C12—H12B	108.1
C3—C4—C5	119.6 (4)	H12A—C12—H12B	107.3
C3—C4—H4	120.2	C12—C13—H13A	109.5
C5—C4—H4	120.2	C12—C13—H13B	109.5
C6—C5—C4	118.7 (4)	H13A—C13—H13B	109.5
C6—C5—H5	120.6	C12—C13—H13C	109.5
C4—C5—H5	120.6	H13A—C13—H13C	109.5
N1—C6—C5	122.4 (4)	H13B—C13—H13C	109.5
N1—C6—C7	115.8 (3)	N11—C14—C15	113.7 (4)
C5—C6—C7	121.8 (4)	N11—C14—H14A	108.8
N8—C7—C6	122.0 (4)	C15—C14—H14A	108.8
N8—C7—H7	119	N11—C14—H14B	108.8
C6—C7—H7	119	C15—C14—H14B	108.8
C7—N8—C9	122.0 (4)	H14A—C14—H14B	107.7
C7—N8—Hg1	120.6 (3)	C14—C15—H15A	109.5
C9—N8—Hg1	117.2 (3)	C14—C15—H15B	109.5
N8—C9—C10	108.7 (4)	H15A—C15—H15B	109.5
N8—C9—H9A	109.9	C14—C15—H15C	109.5
C10—C9—H9A	109.9	H15A—C15—H15C	109.5
N8—C9—H9B	109.9	H15B—C15—H15C	109.5
